# Variation in Breast Cancer Subtype Incidence and Distribution by Race/Ethnicity in the United States From 2010 to 2015

**DOI:** 10.1001/jamanetworkopen.2020.20303

**Published:** 2020-10-19

**Authors:** Xiangyi Kong, Zhiqiang Liu, Ran Cheng, Li Sun, Shaolong Huang, Yi Fang, Jing Wang

**Affiliations:** 1 Department of Breast Surgical Oncology, National Cancer Center, National Clinical Research Center for Cancer, Cancer Hospital, Chinese Academy of Medical Sciences and Peking Union Medical College, Chaoyang District, Beijing, China; 2 Department of Radiotherapy, National Cancer Center, National Clinical Research Center for Cancer, Cancer Hospital, Chinese Academy of Medical Sciences and Peking Union Medical College, Chaoyang District, Beijing, China; 3 Wolfson Institute of Preventive Medicine, Barts Cancer Institute, Queen Mary University of London Charterhouse Square, London, United Kingdom; 4 Department of Breast Surgery, Tongren Municipal People’s Hospital, Tongren, Guizhou, China

## Abstract

**Importance:**

Breast cancer is the most commonly diagnosed cancer and the leading cause of death in women worldwide. Yet the racial/ethnic disparity in incidences and distributions of breast cancer remains largely unknown.

**Objective:**

To examine the racial/ethnic patterns associated with the incidence of the subtypes of breast cancer and distribution of patients across clinicopathological variables.

**Design, Setting, and Participants:**

This population-based cohort study analyzed data from the Surveillance, Epidemiology, and End Results (SEER) database, which collected data from 18 SEER cancer registries that identified patients with breast cancer in the US diagnosed between January 1, 2010, and December 31, 2015. The inclusion criteria were (1) female patients with primary unilateral breast cancer who underwent surgical treatment; (2) record of estrogen receptor, progesterone receptor, and ERBB2 status; (3) record of medical history and histological subtype of the specified tumor location; and (4) data on patient race/ethnicity, lateral tumor position, tumor size, tumor TNM stage, and number of tumors. Incidence and distribution rates were identified and compared for different molecular subtypes, histological grades, pathological patterns, T stages, TNM stages, and tumor sites of breast cancers for each racial/ethnic group. Patient race/ethnicity was classified as follows: non-Hispanic White, Hispanic White, Black, Asian/Pacific Islander, American Indian/Alaskan Native, and unknown. Data were analyzed from January 1, 2010, to December 31, 2015.

**Main Outcomes and Measures:**

Incidence rates of 4 molecular subtypes: hormone receptor (HR)–positive and ERBB2-negative, HR-positive and ERBB2-positive, HR-negative and ERBB2-positive, and triple-negative breast cancer (TNBC). Data on distribution by histological grades (grades 1-4 and unknown), pathological patterns, T stages, TNM stages, and tumor sites was also extracted. Annual age-standardized incidence rates and incidence rate ratios (IRRs) were calculated with 95% CIs. Race/ethnicity case-to-case odds ratios were estimated using polytomous regression.

**Results:**

A total of 239 211 women with breast cancer were analyzed (median [interquartile range]) age, 60 [50-69] years). The annual incidence rate of all breast cancers was 31.3 (95% CI, 31.2- 31.5) per 100 000 people in non-Hispanic White women (the reference group), which was higher compared with the incidence among Black women (IRR, 1.04; 95% CI, 1.02-1.05; *P* < .001). The incidence rates were also lower in Asian/Pacific Islander (IRR, 0.90; 95% CI, 0.89-0.92; *P* < .001), American Indian/Alaskan native (IRR, 0.82; 95% CI, 0.81-0.83; *P* < .001), and Hispanic White women (IRR, 0.79; 95% CI, 0.75-0.83; *P* < .001). In Black patients, the incidences of the HR-positive and ERBB2-positive (IRR, 1.12; 95% CI, 1.08-1.16; *P* < .001), HR-negative and ERBB2-positive (IRR, 1.46; 95% CI, 1.38-1.54; *P* < .001), and TNBC (IRR, 2.07; 95% CI, 2.01-2.14; *P* < .001) subtypes were higher than those in non-Hispanic White patients, but the incidence of the HR-positive and ERBB2-negative subtype in Black women was lower (IRR, 0.86; 95% CI, 0.84-0.87; *P* < .001). The incidences of histological grade 1 (IRR, 0.75; 95% CI, 0.73-0.78; *P* < .001) and grade 2 (IRR, 0.91; 95% CI, 0.89-0.93; *P* < .001) were lower in Asian/Pacific Islander vs non-Hispanic White patients. Non-Hispanic White patients had a higher proportion of lobular carcinoma (9.7% [n = 15 718]) and tubular adenocarcinoma (0.6% [n = 997]) than Black (7.2% [n = 1933]; 0.3% [n = 81]), Asian/Pacific Islander (5.7% [n = 1202]; 0.3% [n = 55]), Hispanic White (7.2% [n = 1985]; 0.3% [n = 88]), and American Indian/Alaskan Native patients (7.2% [n = 101]; 0.4% [n = 5]).

**Conclusions and Relevance:**

This cohort study found notable disparities in incidences and proportions of different molecular subtypes, histological grades, pathological patterns, T stages, TNM stages, and tumor sites associated with race/ethnicity. The findings suggest that combining epidemiologic with genomic and molecular profiling data warrants further research.

## Key Points

**Question:**

Are there variations in the incidence of breast cancer subtypes and the distribution of patients across clinicopathological variables associated with race/ethnicity?

**Findings:**

In this cohort study of 239 211 women with breast cancer, the incidence of HR-negative and ERBB2 (formerly HER2)–positive, HR-positive and ERBB2-positive, and triple-negative breast cancer was higher in Black women compared with non-Hispanic White women. The incidence of histological grade 1 and 2 cancer was lower in Asian/Pacific Islander than non-Hispanic White patients, and a lower incidence of infiltrating duct carcinoma, lobular carcinoma, and tubular adenocarcinoma was seen in Hispanic White vs non-Hispanic White women.

**Meaning:**

Results of this study suggest that combining epidemiologic data with genomic and molecular profiling data may serve as a future research direction and help in closing the gap in breast cancer subtype incidences and distributions associated with race/ethnicity.

## Teaser

This cohort study describes the incidence rates of 4 breast cancer molecular subtypes stratified by race/ethnicity among women in the US.

## Introduction

Breast cancer is the most commonly diagnosed cancer and the leading cause of death in women worldwide.^1,2^ Despite recent advances in treatment and prognosis of this disease, the racial/ethnic gap in incidence and death rates has widened. In the United States, the incidence of breast cancer is higher among Black and White individuals younger than 45 years, whereas the rate in White women between the ages of 60 and 84 years is substantially higher than in Black women.^3^ Although the incidence in Black women is lower, the mortality rate is higher in Black women than in White women.^4,5^ A propensity-matched study using the National Cancer Data Base reported that, among female patients with hormone receptor (HR)–positive breast cancer, Black women had twice the risk of death compared with White women.^6^ Miller et al^7^ reported that, in a study of more than 1.3 million women in the US who were diagnosed with breast cancer between 2001 and 2009, the survival rate for Black women was consistently 10 percentage points lower than for White women, and this disparity persisted over time.

The racial/ethnic disparity in breast cancer is complex, comprising biological heterogeneity; the presence of comorbid conditions; and the multifaceted interactions of behavioral and social factors as well as access to quality health care.^8,9^ Dismantling the biological and access factors associated with the death rate in breast cancer is essential for the development of successful measures. Emerson et al^10^ used data from the Carolina Breast Cancer Study 3 involving 2998 women with invasive breast cancer to evaluate the interaction between race/ethnicity and tumor characteristics; the study oversampled younger women (<50 years) and Black women. The authors used latent class analysis to reduce the size of the data and to capture complex patterns, which varied across people from different racial/ethnic groups. As a result, compared with White women, Black women were found to have lower socioeconomic status, higher cure barriers, and multiple aggressive aggregation of tumors.^10^

Warner et al^11^ examined 20 025 female patients with breast cancer at the National Comprehensive Cancer Network (NCCN) centers to identify the race/ethnicity-specific survival rates based on subtypes and the mediating factors in breast cancer incidence and survival. Although this study by Warner et al^11^ was well done, it analyzed limited data and subtype dimensions. Apart from the molecular subtype, race/ethnicity-based disparities in breast cancer might also be associated with the histological grades, pathological patterns, T stages, TNM stages (per the American Joint Committee on Cancer [AJCC] *Cancer Staging Manual*), and tumor sites. Furthermore, 5 years have passed since the study by Warner et al.^11^ The genetic characteristics of patient populations, the socioeconomic status associated with different racial/ethnic groups, and the available comprehensive treatments (including surgical procedure, radiotherapy, chemotherapy, targeted therapy, endocrine therapy, and immunotherapy) constantly change.

The racial/ethnic disparity in the incidence and distribution of breast cancer is still largely unknown. Analysis of this problem with a wider perspective and a larger sample size is necessary. In this cohort study, we used the Surveillance, Epidemiology, and End Results (SEER) Program to identify rates of breast cancer in the United States by molecular subtypes, histological grades, pathological variations, tumor size, TNM stages, and tumor locations among patients from different race/ethnic groups. We aimed to examine the racial/ethnic patterns associated with the incidence of the subtypes of breast cancer and distribution of patients across clinicopathological variables.

## Methods

This cohort study was approved by SEER, and the requirement for informed patient consent was waived based on the Common Rule. Each study procedure complied with the Declaration of Helsinki^12,13^ or similar standards of ethics. We followed the Strengthening the Reporting of Observational Studies in Epidemiology (STROBE) reporting guideline.

### Study Population

Data were obtained from the SEER database, which collected data from 18 SEER cancer registries that identified all US patients diagnosed with breast cancer from January 1, 2010, to December 31, 2015. The SEER Program of the National Cancer Institute collects information on patients with cancer, including sociodemographic data, tumor and histological sites, and clinical outcomes; it captures approximately 97% of the incident cancers within the catchment area zones, covering approximately 28% of the US population.^14^ Because the relative breast cancer survival rate was more than 90%, data on the long-term follow-up of patients with breast cancer were collected. In addition, we included follow-up information for patients diagnosed in 2010 because recording of ERBB2 (formerly known as HER2) status began in 2010.

We used the following criteria for inclusion in this analysis: (1) female patients with primary unilateral breast cancer who underwent surgical treatment; (2) record of estrogen receptor, progesterone receptor, and ERBB2 status; (3) record of medical history and histological subtype of the specified tumor location; and (4) data on patient race/ethnicity, lateral tumor position, tumor size, tumor TNM stage (per the AJCC seventh edition), and number of tumors. We excluded patients who did not meet these criteria.

The SEER Program uses the self-identified race/ethnicity information in the US Census (the 2000 Census for the study period we analyzed) and on death certificates.^14^ In this study, the race/ethnicity classifications were non-Hispanic White, Hispanic White, Black, Asian/Pacific Islander, American Indian/Alaskan Native, and unknown. Persons of unknown race/ethnicity were excluded from the primary statistical analysis.

### Study Variables and End Points

The SEER database identified breast cancer subtypes by immunohistochemistry HR and ERBB2 status. Other variables that we extracted from the selected cohorts included age and year at diagnosis, histological grades (1-4 and unknown), pathological patterns (infiltrating duct carcinoma [SEER histological type: 8500/3], lobular carcinoma [SEER histological type: 8520/3], infiltrating duct and lobular carcinoma [SEER histological type: 8522/3], cribriform carcinoma [SEER histological type: 8201/3], tubular adenocarcinoma [SEER histological type: 8211/3], mucinous adenocarcinoma [SEER histological type: 8480/3], infiltrating duct mixed with other types of carcinoma [SEER histological type: 8523/3], ductal carcinoma micropapillary [SEER histological type: 8507/3], and others), tumor size, TNM stages, and tumor sites.

### Statistical Analysis

To calculate age-standardized incidence rates and incidence rate ratios (IRRs) with 95% CIs, we used SEER*Stat, version 8.3.6 software (National Cancer Institute). We adopted 2-sided tests to match the race/ethnicity of patients with subtypes of breast cancer. To calculate case to case odds ratios (ORs) for racial/ethnic groups, we used polytomous logistic regression compared with the non-Hispanic White group (the reference group), adjusting for age, geographical region, and year of diagnosis. As the comparison outcome—for example, if the HR-positive and ERBB2-negative subtype was identified—the case-to-case ORs were equal to a modified ratio for the subtype of breast cancer and the race/ethnicity of a subpopulation.

A 2-sided *P* < .05 or an IRR whose 95% CI excluded 1.0 was considered statistically significant. Data were analyzed from January 1, 2010, to December 31, 2015.

## Results

A total of 239 211 patients were diagnosed with breast cancer between 2010 and 2015 and included in this study. This cohort was composed of 162 359 non-Hispanic White (67.9%), 1403 American Indian/Alaskan Native (0.6%), 26 938 Black (11.3%), 27 425 Hispanic White (11.5%), and 21 086 Asian/Pacific Islander (8.8%) women. The median (interquartile range [IQR]) age of these patients was 60 (50-69) years, and the median (IQR) age varied by breast cancer subtype: 61 (51-70) years for women with HR-positive and ERBB2-negative subtype, 56 (47-66) years for women with HR-positive and ERBB2-positive subtype, 56 (48-65) years for women with HR-negative and ERBB2-positive subtype, and 57 (48-67) years for women with triple-negative breast cancer (TNBC) subtype. Most non-Hispanic White patients (n = 90 752 [55.9%]) were 60 years or older, whereas most Black (15 045 [55.9%]), Asian/Pacific Islander (12 261 [58.1%]), Hispanic White (17 109 [62.4%]), and American Indian/Alaskan Native (761 [54.2%]) patients were younger than 60 years (Table). More diagnoses were recorded in later years than in earlier years of the study period except for the last year of study for American Indian/Alaskan Native patients.

**Table.** Select Characteristics of Patients With Breast Cancer, Stratified by Race/Ethnicity

Characteristic	No. (%)				
	Non-Hispanic White (n = 162 359)	Black (n = 26 938)	Asian/Pacific Islander (n = 21 086)	Hispanic White (n = 27 425)	American Indian/Alaskan Native (n = 1403)
Age at diagnosis, y					
<40	7094 (4.4)	2092 (7.8)	1661 (7.9)	2729 (10.0)	106 (7.6)
40-44	9446 (5.8)	2121 (7.9)	2142 (10.2)	2939 (10.7)	99 (7.1)
45-49	15 337 (9.4)	3164 (11.7)	2804 (13.3)	3894 (14.2)	160 (11.4)
50-54	19 212 (11.8)	3790 (14.1)	2898 (13.7)	3958(14.4)	200(14.3)
55-59	20 518 (12.6)	3878 (14.4)	2756 (13.1)	3589 (13.1)	196 (14.0)
60-64	23 141 (14.3)	3644 (13.5)	2813 (13.3)	3173 (11.6)	217 (15.5)
65-69	22 548 (13.9)	2986 (11.1)	2320 (11.0)	2764 (10.1)	181 (12.9)
70-74	17 055(10.5)	2128 (7.9)	1577 (7.5)	1854 (6.8)	120 (8.6)
75-79	12 246 (7.5)	1527 (5.7)	977 (4.6)	1229 (4.5)	78 (5.6)
≥80	15 762 (9.7)	1608 (6.0)	1138 (5.4)	1296 (4.7)	46 (3.3)
Diagnosis year					
2010	24 562 (15.1)	3896 (14.5)	2944 (14.0)	3639 (13.3)	197 (14.0)
2011	25 948 (16.0)	4158 (15.4)	3223 (15.3)	4265 (15.6)	238 (17.0)
2012	26 819 (16.5)	4507 (16.7)	3357 (15.9)	4489 (16.4)	239 (17.0)
2013	27 519 (16.9)	4597 (17.1)	3671 (17.4)	4753 (17.3)	242 (17.2)
2014	28 298 (17.4)	4777 (17.7)	3802 (18.0)	4905 (17.9)	244 (17.4)
2015	29 213 (18.0)	5003 (18.6)	4089 (19.4)	5374 (19.6)	243 (17.3)
SEER region					
West^a^	74 049 (45.6)	6264 (23.3)	16952 (80.4)	22381 (81.6)	1267 (90.3)
Other^b^	88 310 (54.4)	20674 (76.7)	4134 (19.6)	5044 (18.4)	136 (9.7)

Abbreviation: SEER, Surveillance, Epidemiology, and End Results program.

^a^ West region includes registries from Hawaii, New Mexico (and Arizona American Indians), Puget Sound/Seattle, Utah, Alaskan Natives, and California.

^b^ Other region includes registries from Detroit, Michigan; Iowa; Connecticut; New Jersey; Atlanta and Rural Georgia; Kentucky; and Louisiana.

### Incidence of Breast Cancer by Race/Ethnicity

The annual incidence rate of all breast cancers in non-Hispanic White women was 31.3 (95% CI, 31.2- 31.5) per 100 000 people, which was higher compared with the incidence among Black women (IRR, 1.04; 95% CI, 1.02-1.05; *P* < .001). The incidence rates were also lower in Asian/Pacific Islander (IRR, 0.90; 95% CI, 0.89-0.92; *P* < .001), American Indian/Alaskan Native (IRR, 0.82; 95% CI, 0.81- 0.83; *P* < .001), and Hispanic White (IRR, 0.79; 95% CI, 0.75-0.83; *P* < .001) women.

#### Tumor Molecular Subtype and Histological Grade 

**Figure 1.**
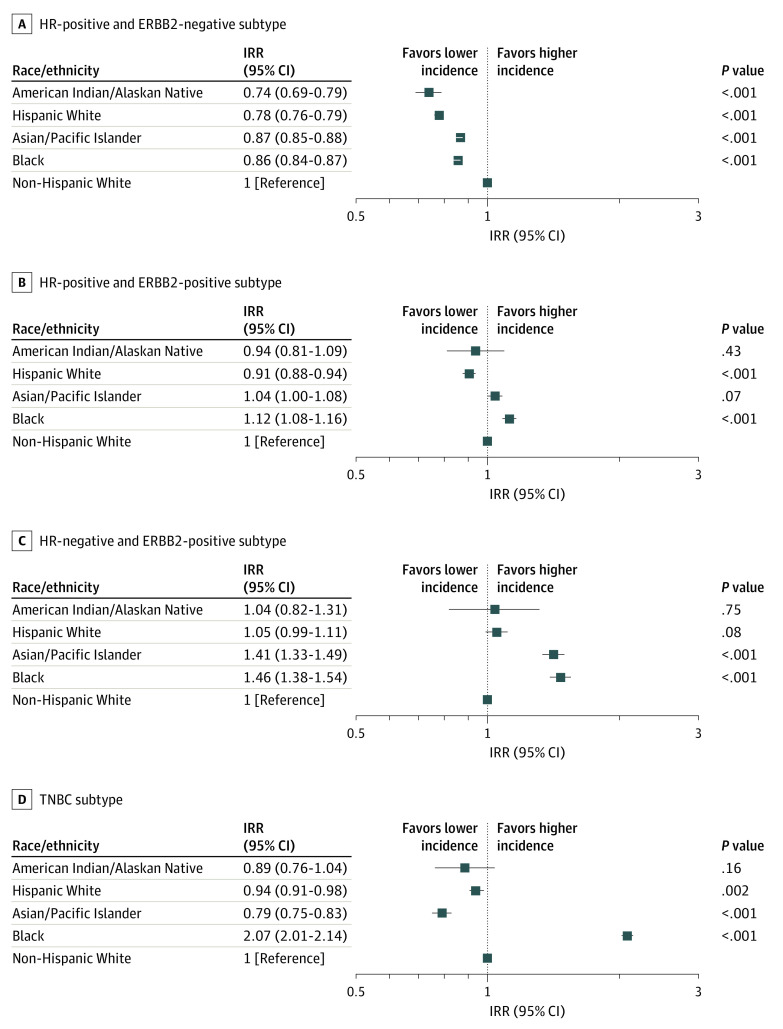
Incidence Rate Ratio (IRR) of Breast Cancer Molecular Subtypes by Race/Ethnicity Compared With Non-Hispanic White Individuals, 2010-2015 The IRRs were calculated from age-standardized incidence rates. The squares represent the IRR values and the horizontal lines represent the 95% CIs. HR indicates hormone receptor; TNBC, triple-negative breast cancer.

The incidence of HR-positive and ERBB2-negative, HR-negative and ERBB2-positive, HR-positive and ERBB2-positive, and TNBC molecular subtypes varied markedly according to race/ethnicity (Figure 1). In Black patients, the incidences of HR-positive and ERBB2-positive (IRR, 1.12; 95% CI, 1.08-1.16; *P* < .001), HR-negative and ERBB2-positive (IRR, 1.46; 95% CI, 1.38-1.54; *P* < .001), and TNBC (IRR, 2.07; 95% CI, 2.01-2.14; *P* < .001) subtypes were higher than those in non-Hispanic White patients, but the incidence of HR-positive and ERBB2-negative subtype in Black women was lower (IRR, 0.86; 95% CI, 0.84-0.87; *P* < .001). Compared with non-Hispanic White women, Asian/Pacific Islander women had a higher incidence of HR-negative and ERBB2-positive subtype (IRR, 1.41; 95% CI, 1.33-1.49; *P* < .001) but lower incidences of HR-positive and ERBB2-negative (IRR, 0.87; 95% CI, 0.85-0.88; *P* < .001) and TNBC (IRR, 0.79; 95% CI, 0.75-0.83; *P* < .001) subtypes. Hispanic White patients had lower incidences of HR-positive and ERBB2-negative (IRR, 0.78; 95% CI,0.76-0.79; *P* < .001), HR-positive and ERBB2-positive (IRR, 0.91; 95% CI, 0.88-0.94; *P* < .001), and TNBC (IRR, 0.94; 95% CI, 0.91-0.98; *P* = .002) subtypes than non-Hispanic White patients. The incidence of HR-positive and ERBB2-negative subtype (IRR, 0.74; 95% CI, 0.69-0.79; *P* < .001) among American Indian/Alaskan Native women was lower than in their non-Hispanic White counterparts.

**Figure 2.**
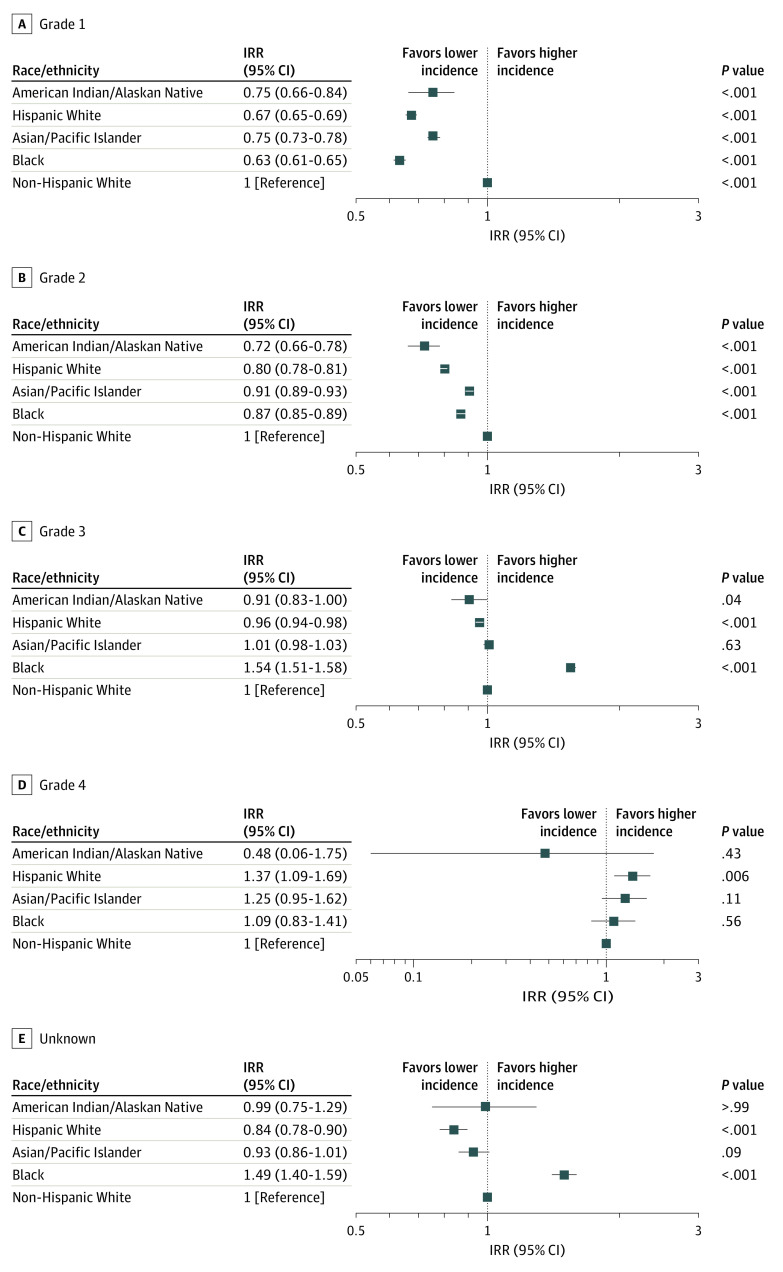
Incidence Rate Ratio (IRR) of Breast Cancer Histological Grades by Race/Ethnicity Compared With Non-Hispanic White Individuals, 2010-2015 The IRRs were calculated from age-standardized incidence rates. The squares represent the IRR values and the horizontal lines represent the 95% CIs.

The frequency of various histological grades of breast cancer varied statistically significantly by race/ethnicity (Figure 2). In Black patients, the incidences of grade 1 (IRR, 0.63; 95% CI, 0.61-0.65; *P* < .001) and grade 2 (IRR, 0.87; 95% CI, 0.85-0.89; *P* < .001) cancers were lower than in non-Hispanic white patients, but grade 3 incidence was higher (IRR, 1.54; 95% CI, 1.51-1.58; *P* < .001). No statistically significant difference was detected for grade 4 (IRR, 1.09; 95% CI, 0.83-1.41; *P* = .56). The incidences of grade 1 (IRR, 0.75; 95% CI, 0.73-0.78; *P* < .001) and grade 2 cancers (IRR, 0.91; 95% CI, 0.89-0.93; *P* < .001) were lower in Asian/Pacific Islander vs non-Hispanic White patients. For grade 3 (IRR, 1.01; 95% CI, 0.98-1.03; *P* = .63) and grade 4 (IRR, 1.25; 95% CI, 0.95-1.62; *P* = .11) incidences, no marked difference was found between the 2 patient groups. Compared with non-Hispanic White women, Hispanic White women had lower grade 1 (IRR, 0.67; 95% CI, 0.65-0.69; *P* < .001), grade 2 (IRR, 0.80; 95% CI, 0.78-0.81; *P* < .001), and grade 3 (IRR, 0.96; 95% CI, 0.94-0.98; *P* < .001) incidences. The incidences of grade 1 (IRR, 0.75; 95% CI, 0.66-0.84; *P* < .001) and grade 2 cancers (IRR, 0.72; 95% CI, 0.66-0.78; *P* < .001) among American Indian/Alaskan Native patients were lower than in non-Hispanic White patients.

#### Tumor Pathological Pattern 

The incidence of different patterns of pathological breast cancer also statistically significantly varied by race/ethnicity (eFigure 1 in the Supplement). In Black women, the incidences of lobular carcinoma (IRR, 0.81; 95% CI, 0.78-0.85; *P* < .001), infiltrating duct and lobular carcinoma (IRR, 0.68; 95% CI, 0.63-0.72; *P* < .001), and tubular adenocarcinoma (IRR, 0.51; 95% CI, 0.40-0.64; *P* < .001) were lower compared with those among non-Hispanic White women, whereas the incidences of infiltrating duct carcinoma (IRR, 1.06; 95% CI, 1.05-1.08; *P* < .001), mucinous adenocarcinoma (IRR, 1.34; 95% CI, 1.22-1.47; *P* < .001), and ductal carcinoma micropapillary (IRR, 1.26; 95% CI, 1.03-1.54; *P* = .03) were higher. No statistically significant difference in incidence was found for cribriform carcinoma (IRR, 0.90; 95% CI, 0.63-1.26; *P* = .60). In Asian/Pacific Islander patients, the incidences of infiltrating duct carcinoma (IRR, 0.95; 95% CI, 0.94-0.97; *P* < .001), lobular carcinoma (IRR, 0.55; 95% CI, 0.52-0.59; *P* < .001), infiltrating duct and lobular carcinoma (IRR, 0.72; 95% CI, 0.67-0.76; *P* < .001), and tubular adenocarcinoma (IRR, 0.39; 95% CI, 0.29-0.51; *P* < .001) were lower compared with non-Hispanic White patients, whereas the incidence of mucinous adenocarcinoma (IRR, 1.31; 95% CI, 1.18-1.44; *P* < .001) was higher. No statistically significant differences in incidences were noted for cribriform carcinoma (IRR, 1.08; 95% CI, 0.76-1.50; *P* = .68), infiltrating duct mixed with other types of carcinoma (IRR, 0.96; 95% CI, 0.89-1.04; *P* = .32), and ductal carcinoma micropapillary (IRR, 1.15; 95% CI, 0.92-1.43; *P* = .22) between Asian/Pacific Islander and non-Hispanic White patients.

Hispanic White patients vs their non-Hispanic White counterparts had lower incidences of infiltrating duct carcinoma (IRR, 0.82; 95% CI, 0.81-0.84; *P* < .001), lobular carcinoma (IRR, 0.68; 95% CI, 0.64-0.71; *P* < .001), infiltrating duct and lobular carcinoma (IRR, 0.90; 95% CI, 0.86-0.95; *P* < .001), tubular adenocarcinoma (IRR, 0.44; 95% CI, 0.35-055; *P* < .001), and infiltrating duct mixed with other types of carcinoma (IRR, 0.90; 95% CI, 0.84-0.97; *P* = .005). The incidences of infiltrating duct carcinoma (IRR, 0.83; 95% CI, 0.78-0.88; *P* < .001), lobular carcinoma (IRR, 0.61; 95% CI, 0.49-0.75; *P* < .001), tubular adenocarcinoma (IRR, 0.15; 95% CI, 0.00-0.69; *P* = .006), mucinous adenocarcinoma (IRR, 0.54; 95% CI, 0.30-0.91; *P* = .02), and infiltrating duct mixed with other types of carcinoma (IRR, 0.59; 95% CI, 0.39-0.84; *P* = .002) among American Indian/Alaskan Native women were lower than among non-Hispanic White women.

#### Tumor Size and TNM Stage 

**Figure 3.**
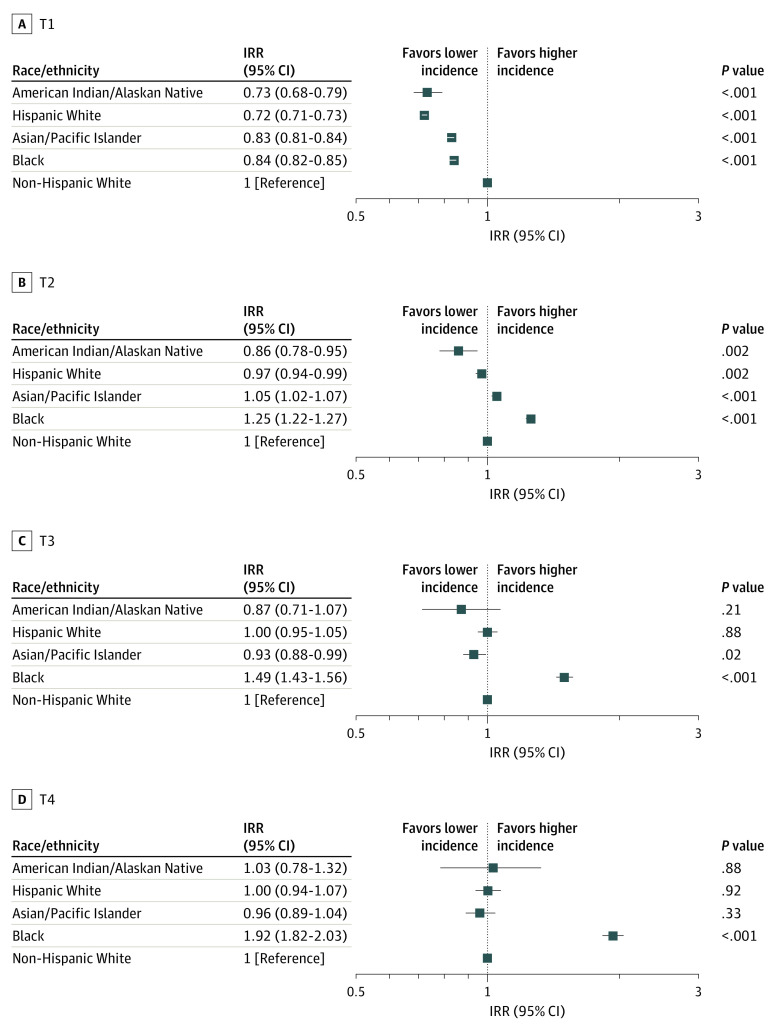
Incidence Rate Ratio (IRR) of Breast Cancer Tumor Size by Race/Ethnicity Compared With Non-Hispanic White Individuals, 2010-2015 The IRRs were calculated from age-standardized incidence rates. The squares represent the IRR values and the horizontal lines represent the 95% CIs.

The incidence of different sizes of breast tumors varied considerably by race/ethnicity (Figure 3). In Black patients, the incidence of T1 (IRR, 0.84; 95% CI, 0.82-0.85; *P* < .001) was lower than in non-Hispanic White patients, whereas T2 (IRR, 1.25; 95% CI, 1.22-1.27; *P* < .001), T3 (IRR, 1.49; 95% CI, 1.43-1.56; *P* < .001), and T4 (IRR, 1.92; 95% CI, 1.82-2.03; *P* < .001) incidences were higher. Among Asian/Pacific Islander patients, the incidences of T1 (IRR, 0.83; 95% CI, 0.81-0.84; *P* < .001) and T3 (IRR, 0.93; 95% CI, 0.88-0.99; *P* = .02) were smaller, and the incidence of T2 (IRR, 1.05; 95% CI, 1.02-1.07; *P* < .001) was higher compared with non-Hispanic White patients. No marked difference in T4 incidence was observed in Asian/Pacific Islander patients (IRR, 0.96; 95% CI, 0.89-1.04; *P* = .33). Hispanic White women had lower incidences of T1 (IRR, 0.72; 95% CI, 0.71-0.73; *P* < .001) and T2 (IRR, 0.97; 95% CI, 0.94-0.99; *P* < .002) than non-Hispanic White women. The incidences of T1 (IRR, 0.73; 95% CI, 0.68-0.79; *P* < .001) and T2 (IRR, 0.86; 95% CI, 0.78-0.95; *P* = .002) among American Indian/Alaskan Native women were also lower than among their non-Hispanic White counterparts.

**Figure 4.**
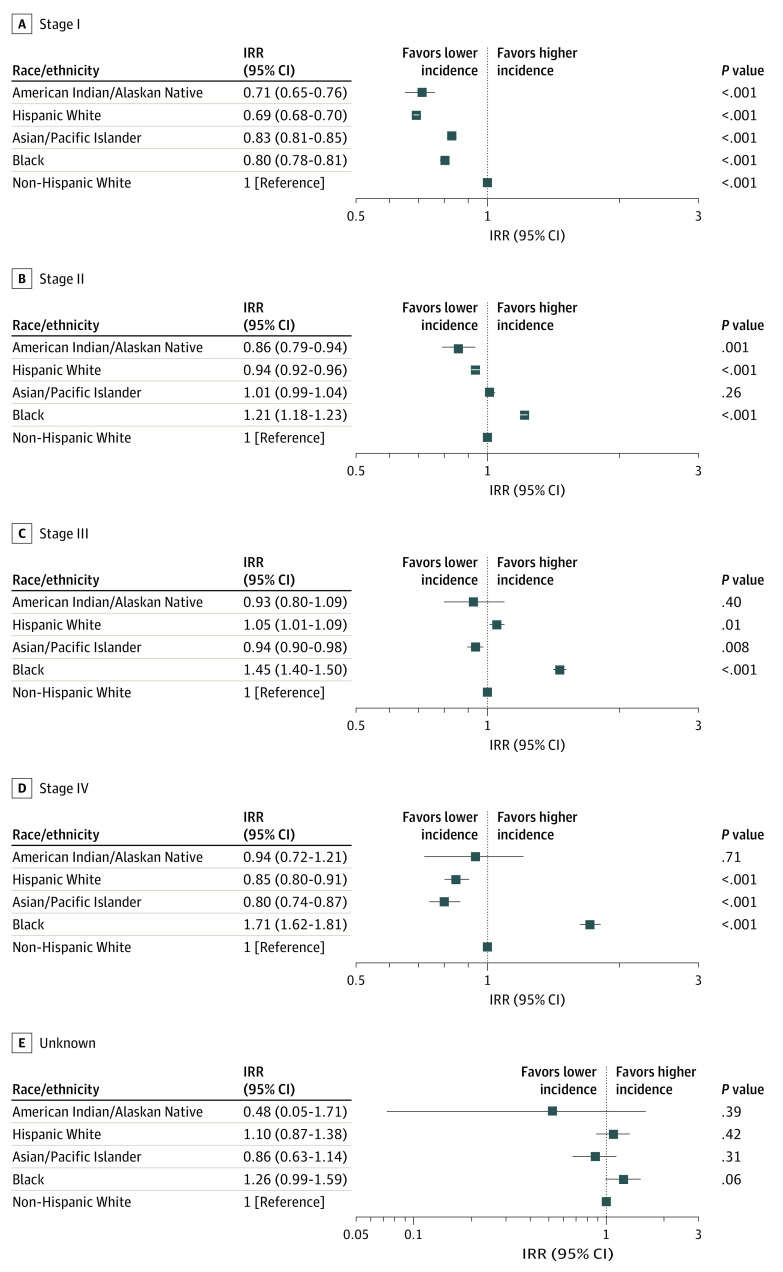
Incidence Rate Ratio (IRR) of the TNM Stages of Breast Cancer by Race/Ethnicity Compared With Non-Hispanic White Individuals, 2010-2015 The IRRs were calculated from age-standardized incidence rates. The squares represent the IRR values and the horizontal lines represent the 95% CIs.

With respect to the AJCC TNM stages (Figure 4), the incidence of stage I cancer (IRR, 0.80; 95% CI, 0.78-0.81; *P* < .001) was lower in Black patients compared with non-Hispanic White patients, whereas stage II (IRR, 1.21; 95% CI, 1.18-1.23; *P* < .001), stage III (IRR, 1.45; 95% CI, 1.40-1.50; *P* < .001), and stage IV tumor (IRR, 1.71; 95% CI, 1.62-1.81; *P* < .001) incidences were higher. The incidences of stage I (IRR, 0.83; 95% CI, 0.81-0.85; *P* < .001), stage III (IRR, 0.94; 95% CI, 0.90-0.98; *P* = .008), and stage IV tumors (IRR, 0.80; 95% CI, 0.74-0.87; *P* < .001) were lower among Asian/Pacific Islander women; stage II incidence was not statistically significantly different compared with non-Hispanic White women (IRR, 1.01; 95% CI, 0.99-1.04; *P* = .26). Hispanic White vs non-Hispanic White women had lower incidences of stage I (IRR, 0.69; 95% CI, 0.68-0.70; *P* < .001), stage II (IRR, 0.94; 95% CI, 0.92-0.96; *P* < .001), and stage IV tumors (IRR, 0.85; 95% CI, 0.80-0.91; *P* < .001) as well as a higher incidence of stage III tumors (IRR 1.05; 95% CI, 1.01-1.09; *P* = .01). The incidences of stage I (IRR, 0.71; 95% CI, 0.65-0.76; *P* < .001) and stage II tumors (IRR, 0.86; 95% CI, 0.79-0.94; *P* < .001) among American Indian/Alaskan Native women were also lower than those in non-Hispanic White women.

The incidence of breast tumors in different locations also varied by race/ethnicity (eFigure 2 in the Supplement). Tumors in the inner quadrant were higher among Black women than in non-Hispanic White women (upper inner quadrant: IRR, 1.04 [95% CI, 1.01-1.08; *P* = .02]; lower inner quadrant: IRR, 1.18 [95% CI, 1.12-1.24; *P* < .001]).

### Distribution of Patients by Race/Ethnicity

#### Tumor Molecular Subtype and Histological Grade 

The proportion of Black patients with the HR-positive and ERBB2-negative subtype was smaller than in non-Hispanic White patients (16 372 [60.8%] vs 122 295 [75.3%]) (eTable 1 in the Supplement). Compared with non-Hispanic White patients (16 902 [10.4%] and 6739 [4.2%]), more Asian/Pacific Islander (2660 [12.6%] and 1436 [6.8%]), Hispanic White (3482 [12.7%] and 1585 [5.8%]), and American Indian/Alaskan Native (188 [13.4%] and 80 [5.7%]) patients had both HR-positive and ERBB2-positive subtype and HR-negative and ERBB2-positive subtype diagnoses. Approximately 10% of non-Hispanic White (16 423 [10.1%]), Asian/Pacific Islander (1923 [9.1%]), Hispanic White (3486 [12.7%]), and American Indian/Alaskan Native (166 [11.8%]) women had the TNBC subtype compared with approximately 21% of Black patients (5713 [21.2%]). Pairwise tests of the number of molecular subtypes between racial/ethnic groups demonstrated great disparities for all pairs of race/ethnic subgroups (all *P* < .001) except for Hispanic White women compared with American Indian/Alaskan Native women.

Histological grade 2 (moderately differentiated) breast cancer was the most common among non-Hispanic White (70 336 [43.3%]), Asian/Pacific Islander (9091 [43.1%]), Hispanic White (11 222 [40.9%]), and American Indian/Alaskan Native (558 [39.8%]) patients (eTable 2 in the Supplement). For Black patients, the most common breast cancer was grade 3 (poorly differentiated), which was reported in 12 177 patients (45.2%) and with a case-to-case OR of 2.4 (95% CI, 2.30-2.49).

Black patients had a higher histological grade than patients of other race/ethnicity. A lower percentage of Asian/Pacific Islander (19.5% [n = 4102]), Hispanic White (18.2% [n = 4979]), and American Indian/Alaskan Native (21.1% [n = 296]) patients were diagnosed with grade 1 breast cancer than their non-Hispanic White counterparts (24.2% [n = 39 267]). A lower percentage of Asian/Pacific Islander (43.1% [n = 9091]) and Hispanic White (40.9% [n = 11 222]) patients were diagnosed with grade 2 breast cancer compared with non-Hispanic White patients (43.3% [n = 70 336]); however, the difference in percentages between American Indian/Alaskan Native and non-Hispanic White women was not significant. As mentioned, Black women had the highest percentage of grade 3 tumors among all groups. For grade 4, no difference in percentage was found between Asian/Pacific Islander (0.3% [n = 70]) and non-Hispanic White (0.2% [n = 386]) women, and a higher percentage of Black (0.3% [n = 70]) and Hispanic White (0.4% [n = 117]) women were diagnosed.

#### Tumor Pathological Pattern, TNM Stage, and Size 

Regardless of race/ethnicity, invasive ductal carcinoma was the most common pathological pattern found among patients, followed by lobular carcinoma. Non-Hispanic White patients had a higher proportion of lobular carcinoma (9.7% [n = 15 718]) and tubular adenocarcinoma (0.6% [n = 997]) than Black (7.2% [n = 1933]; 0.3% [n = 81]), Asian/Pacific Islander (5.7% [n = 1202]; 0.3% [n = 55]), Hispanic White (7.2% [n = 1985]; 0.3% [n = 88]), and American Indian/Alaskan Native patients (7.2% [n = 101]; 0.4% [n = 5]). The proportion of patients with cribriform carcinoma, infiltrating duct mixed with other types of carcinoma, and ductal carcinoma micropapillary patterns did not differ statistically significantly by race/ethnicity (eTable 3 in the Supplement).

The proportion of patients with T1-sized tumors (61.2% [n = 99 334]) and TNM stage I breast cancer (53.1% [n = 86 220) were markedly higher in non-Hispanic White patients vs patients of other race/ethnicity (eTables 4 and 5 in the Supplement). The proportion of patients with breast cancer with T2 (29.2% [n = 47 397]) and T3 (5.8% [n = 9466]) tumor size and at stage II (32.3% [n = 52 479]) and stage III (10.4% [n = 16 911]) were statistically significantly lower in non-Hispanic White women compared with Black (T2, 35.9% [n = 9655]; T3, 8.8% [n = 2368]; stage II, 38.4% [n = 10339]; stage III, 15.2% [n = 4084]), Asian/Pacific Islander (T2, 34.5% [n = 7273]; T3, 6.3% [n = 1321]; stage II, 37% [n = 7804]; stage III, 11.2% [n = 2369]), Hispanic White (T2, 36% [n = 9850]; T3, 7.9% [n = 2156]; stage II, 38.9% [n = 10656]; stage III, 14.5% [n = 3970]), and American Indian/Alaskan Native patients (T2, 32.6% [n = 457]; T3, 7% [n = 98]; stage II, 36.1% [n = 506]; stage III, 13% [n = 182]). The difference in the proportion of patients with T3 tumor size was not statistically significant between the non-Hispanic White and Asian/Pacific Islander groups (5.8% [n = 9466] vs 6.3% [n = 1321]). In non-Hispanic White women, the proportion of patients with T4-sized tumors and stage IV breast cancer were statistically significantly lower than in other groups except among Asian/Pacific Islander women (T4: 3.7% [n = 5993] vs 3.8% [n = 800]); stage IV: 3.8% [n = 6241] vs 3.5% [n = 731]).

#### Tumor Primary Site 

The proportion of tumors in different locations in different races also differs statistically significantly (eTable 6 in the Supplement). In all racial/ethnic groups, tumors grew mostly in the upper outer quadrant of the breast, followed by the overlapping quadrant, the upper inner quadrant, and the lower outer quadrant. The proportion of patients with breast tumor in the upper outer quadrant was higher in non-Hispanic White women (35.3% [n = (57 308]) than in Black (34.7% [n = 9337]), Asian/Pacific Islander (32.2% [n = 6789]), Hispanic White (33.7% [n = 9248]), and American Indian/Alaskan Native patients (34.7% [n = 487]). The proportion of patients with a breast tumor in the upper inner quadrant was lower in non-Hispanic White women (12.3% [n = 19 965]) than in Asian/Pacific Islander (14.4% [n = 3041]), Hispanic White (12.5% [n = 3429]), and American Indian/Alaskan Native women (15.1% [n = 212]). The proportion of patients with breast cancer in the central portion or nipple area was statistically significantly lower in Black women (4.2% [n = 1133]) than in non-Hispanic White (5% [n = 8159]), Asian/Pacific Islander (5.7% [n = 1196]), Hispanic White (4.8% [n = 1310]), and American Indian/Alaskan Native women (5.7% [n = 80]).

## Discussion

Breast cancer is the most commonly diagnosed cancer in women worldwide.^2^ It is also the top cause of cancer death in women.^15^ High incidence rates have been found in North America, Australia and New Zealand, Western and Northern Europe, and Asia and Sub-Saharan Africa.^16^ Regional disparities in breast cancer incidence are likely associated with societal changes brought on by industrialization.^16^

Breast cancer is among a diverse group of malignant neoplasms that have characteristics associated with race/ethnicity and region and whose subtype results show substantial variations.^17^ This disease exhibits various morphological characteristics, immunohistochemical profiles, and molecular subtypes with particular clinical course and outcome.^17^ Considering the diversity of the US population and the availability of data from the SEER database, we analyzed the incidences of breast cancer subtypes after diagnosis and distributions of patients across clinicopathological variables.

To our knowledge, this study is the first to carry out a comprehensive yet detailed comparative analysis of breast cancer incidence by race/ethnicity from the perspectives of molecular subtype, histological grade, pathological pattern, tumor size, TNM stage, and tumor location. As an extension of similar previous studies,^18,19,20^ this study also observed the variations in these clinicopathological characters. We believe that this study was an important step toward ameliorating, not just describing, racial/ethnic differences in breast cancer. The results suggest that combining epidemiologic data with genomic and molecular profiling data may be a critical future research direction.

### Strengths and Limitations

This study has some strengths. The most notable strength is the use of the population-based SEER database, which classifies more than 95% of patients with cancer in their geographically dispersed catchment areas, representing approximately 28% of the US population.^21,22^ Hence, the findings of this study reflect current breast cancer incidence in the US. Although Warner et al^11^ previously published a similar study, the non–population-based NCCN database that the authors used only examined patients who had access to and were receiving treatments at key cancer centers. In the Warner et al^11^ report, the median age of patients with breast cancer was nearly 10 years younger than the national median age.

This study has some limitations. The percentage of patients with breast cancer was relatively small because of the relatively small American Indian/Alaskan Native group. Nonetheless, we found statistically significant differences in the proportions and incidence rates in the subtypes of breast cancer. In comparison, misclassifications of race/ethnicity in cancer studies could affect the outcomes; for instance, if the American Indian/Alaskan Native group were misclassified, the group may be mistaken as belonging to the Hispanic White or unknown race group. However, case-to-case ORs with recovery analyses use only medical information and should be less affected by race/ethnicity mismanagement. Also, incidence estimates are sensitive to ascertainment bias and the biases linked to screening and the overdetection of low-grade disease because of differential access to health care or the variation in diagnostic procedure. Thus, incidence rates and IRRs should be interpreted with caution for the American Indian/Alaskan Native group and other racial/ethnic groups.

## Conclusions

In this cohort study, disparities were found in the incidences and proportions of different molecular subtypes, histological grades, pathological patterns, tumor sizes, TNM stages, and tumor sites of breast cancers across different racial/ethnic populations in the United States. This study could serve as a step toward ameliorating the racial/ethnic differences in breast cancer. Future research that combines epidemiologic data with genomic and molecular profiling data may serve as a future research direction and help in closing the race/ethnicity-based gap in the incidence and distribution of breast cancer subtypes.

## Notes

Supplement. **eTable 1.** Number and Percentage of Patients with Each Breast Cancer Molecular Subtype by Race/Ethnic Group and Case-Case ORs for Each Subtype Compared With HR+/HER2- **eTable 2.** Number and Percentage of Patients with Each Breast Cancer Histological Grade by Race/Ethnic Group and Case-Case ORs for Each Grade Compared With Grade I **eTable 3.** Number and Percentage of Patients with Each Breast Cancer Pathological Pattern by Race/Ethnic Group and Case-Case ORs for Each Pattern Compared With Infiltrating Duct Carcinoma **eTable 4.** Number and Percentage of Patients with Each T Stage of Breast Cancer by Race/Ethnic Group and Case-Case ORs for Each T Stage Compared With T1 **eTable 5.** Number and Percentage of Patients with Each AJCC TNM Stage Group of Breast Cancer by Race/Ethnic Group and Case-Case ORs for Each Group Compared With Stage I **eTable 6.** Number and Percentage of Patients with Each Breast Cancer Tumor Site by Race/Ethnic Group and Case-Case ORs for Each Site Compared with Upper-Outer Quadrant of Breast **eFigure 1.** IRR of Breast Cancer Pathological Patterns by Race/Ethnicity Compared With NHWs **eFigure 2.** IRR of Breast Cancer Tumor Locations by Race/Ethnicity Compared With NHWs
