# Supplementary material for: Variation in Breast Cancer Subtype Incidence and Distribution by Race/Ethnicity in the United States From 2010 to 2015
Source: JAMA Netw Open. 2020 Oct 19;3(10):e2020303. doi: 10.1001/jamanetworkopen.2020.20303 (PMC7573683; doi:10.1001/jamanetworkopen.2020.20303)
Supplement: Supplement. — eTable 1. Number and Percentage of Patients with Each Breast Cancer Molecular Subtype by Race/Ethnic Group and Case-Case ORs for Each Subtype Compared With HR+/HER2- eTable 2. Number and Percentage of Patients with Each Breast Cancer Histological Grade by Race/Ethnic Group and Case-Case ORs for Each Grade Compared With Grade I eTable 3. Number and Percentage of Patients with Each Breast Cancer Pathological Pattern by Race/Ethnic Group and Case-Case ORs for Each Pattern Compared With Infiltrating Duct Carcinoma eTable 4. Number and Percentage of Patients with Each T Stage of Breast Cancer by Race/Ethnic Group and Case-Case ORs for Each T Stage Compared With T1 eTable 5. Number and Percentage of Patients with Each AJCC TNM Stage Group of Breast Cancer by Race/Ethnic Group and Case-Case ORs for Each Group Compared With Stage I eTable 6. Number and Percentage of Patients with Each Breast Cancer Tumor Site by Race/Ethnic Group and Case-Case ORs for Each Site Compared with Upper-Outer Quadrant of Breast eFigure 1. IRR of Breast Cancer Pathological Patterns by Race/Ethnicity Compared With NHWs eFigure 2. IRR of Breast Cancer Tumor Locations by Race/Ethnicity Compared With NHWs [file jamanetwopen-e2020303-s001.pdf]

Supplementary Online Content

Kong X, Liu Z, Cheng R, et al. Variation in breast cancer subtype incidence and distribution by race/ethnicity in the United States from 2010 to 2015. *JAMA Netw Open*. 2020;3(10): e2020303. doi:10.1001/jamanetworkopen.2020.20303

eTable 1. Number and Percentage of Patients with Each Breast Cancer Molecular Subtype by Race/Ethnic Group and Case-Case ORs for Each Subtype Compared With HR+/HER2-
eTable 2. Number and Percentage of Patients with Each Breast Cancer Histological Grade by Race/Ethnic Group and Case-Case ORs for Each Grade Compared With Grade I
eTable 3. Number and Percentage of Patients with Each Breast Cancer Pathological Pattern by Race/Ethnic Group and Case-Case ORs for Each Pattern Compared With Infiltrating Duct Carcinoma
eTable 4. Number and Percentage of Patients with Each T Stage of Breast Cancer by Race/Ethnic Group and Case-Case ORs for Each T Stage Compared With T1
eTable 5. Number and Percentage of Patients with Each AJCC TNM Stage Group of Breast Cancer by Race/Ethnic Group and Case-Case ORs for Each Group Compared With Stage I
eTable 6. Number and Percentage of Patients with Each Breast Cancer Tumor Site by Race/Ethnic Group and Case-Case ORs for Each Site Compared with Upper-Outer Quadrant of Breast
eFigure 1. IRR of Breast Cancer Pathological Patterns by Race/Ethnicity Compared With NHWs
eFigure 2. IRR of Breast Cancer Tumor Locations by Race/Ethnicity Compared With NHWs

This supplementary material has been provided by the authors to give readers additional information about their work.

©2020 Kong X et al. *JAMA Network Open*

eTable 1. Number and Percentage of Patients with Each Breast Cancer Molecular Subtype by Race/Ethnic Group and Case-Case ORs for Each Subtype Compared With HR+/HER2-

**Molecular subtype (BreastType)**	**Non-Hispanic White No. (%)**	**Black**			**Asian/Pacific Islander**			**Hispanic White**			**American Indian/Alaskan Native**		
		**No. (%)**	**OR***	**95% CI**	**No. (%)**	**OR***	**95% CI**	**No. (%)**	**OR***	**95% CI**	**No. (%)**	**OR***	**95% CI**
HR+/HER2-	122295 (75.3)	16372 (60.8)	Ref.	-	15067 (71.5)	Ref.	-	18872 (68.8)	Ref.	-	969 (69.1)	Ref.	-
HR+/HER2+	16902 (10.4)	3213 (11.9)	1.28	1.23 to 1.34	2660 (12.6)	1.19	1.14 to 1.25	3482 (12.7)	1.2	1.15 to 1.25	188 (13.4)	1.36	1.16 to 1.59
HR-/HER2+	6739 (4.2)	1640 (6.1)	1.64	1.55 to 1.74	1436 (6.8)	1.66	1.56 to 1.76	1585 (5.8)	1.41	1.33 to 1.5	80 (5.7)	1.47	1.17 to 1.85
Triple Negative	16423 (10.1)	5713 (21.2)	2.4	2.31 to 2.48	1923 (9.1)	0.91	0.87 to 0.96	3486 (12.7)	1.28	1.23 to 1.34	166 (11.8)	1.26	1.07 to 1.49

Abbreviations: NHW, Non-Hispanic White.

*Adjusted for age, SEER registry region, and year of diagnosis. Case-case OR = $(\%_{subtype}/\%_{HR+/HER2-})_{subpop}/(\%_{subtype}/\%_{HR+/HER2-})_{NHW}$.

©2020 Kong X et al. *JAMA Network Open*

eTable 2. Number and Percentage of Patients with Each Breast Cancer Histological Grade by Race/Ethnic Group and Case-Case ORs for Each Grade Compared With Grade I

Histological Grade (Grade)	Non-Hispanic White No. (%)	Black			Asian/Pacific Islander			Hispanic White			American Indian/Alaskan Native		
		No. (%)	OR*	95% CI	No. (%)	OR*	95% CI	No. (%)	OR*	95% CI	No. (%)	OR*	95% CI
Well differentiated; Grade I	39267 (24.2)	3736 (13.9)	Ref.	-	4102 (19.5)	Ref.	-	4979 (18.2)	Ref.	-	296 (21.1)	Ref.	-
Moderately differentiated; Grade II	70336 (43.3)	9646 (35.8)	1.36	1.31 to 1.42	9091 (43.1)	1.26	1.21 to 1.31	11222 (40.9)	1.25	1.21 to 1.3	558 (39.8)	1.09	0.95 to 1.26
Poorly differentiated; Grade III	46819 (28.8)	12177 (45.2)	2.4	2.3 to 2.49	7078 (33.6)	1.4	1.34 to 1.46	10138 (37)	1.56	1.51 to 1.62	488 (34.8)	1.4	1.21 to 1.62
Undifferentiated; anaplastic; Grade IV	386 (0.2)	70 (0.3)	2.14	1.65 to 2.78	70 (0.3)	1.27	0.98 to 1.65	117 (0.4)	1.73	1.4 to 2.15	<=5	NE	
Unknown	5551 (3.4)	1309 (4.9)	2.08	1.94 to 2.23	745 (3.5)	1.63	1.5 to 1.78	969 (3.5)	1.64	1.52 to 1.78	59 (4.2)	1.91	1.44 to 2.53

Abbreviations: NHW, Non-Hispanic White.

*Adjusted for age, SEER registry region, and year of diagnosis. Case-case OR = $(\%_{subtype}/\%_{Grade\ I})_{subpop}/(\%_{subtype}/\%_{Grade\ I})_{NHW}$.

©2020 Kong X et al. *JAMA Network Open*

eTable 3. Number and Percentage of Patients with Each Breast Cancer Pathological Pattern by Race/Ethnic Group and Case-Case ORs for Each Pattern Compared With Infiltrating Duct Carcinoma

Pathological Pattern (HistBehav)	Non-Hispanic White No. (%)	Black			Asian/Pacific Islander			Hispanic White			American Indian/Alaskan Native		
		No. (%)	OR*	95% CI	No. (%)	OR*	95% CI	No. (%)	OR*	95% CI	No. (%)	OR*	95% CI
8500/3: Infiltrating duct carcinoma	121878 (75.1)	21040 (78.1)	Ref.	-	16840 (79.9)	Ref.	-	21111 (77)	Ref.	-	1119 (79.8)	Ref.	-
8520/3: Lobular carcinoma	15718 (9.7)	1933 (7.2)	0.78	0.74 to 0.82	1202 (5.7)	0.58	0.55 to 0.62	1985 (7.2)	0.8	0.76 to 0.84	101 (7.2)	0.72	0.59 to 0.87
8522/3: Infiltrating duct and lobular carcinoma	9387 (5.8)	992 (3.7)	0.66	0.61 to 0.7	964 (4.6)	0.72	0.67 to 0.77	1709 (6.2)	1.04	0.99 to 1.1	83 (5.9)	0.95	0.77 to 1.18
8201/3: Cribriform carcinoma	282 (0.2)	40 (0.1)	0.86	0.62 to 1.19	43 (0.2)	1.28	0.91 to 1.8	48 (0.2)	1.14	0.83 to 1.58	<=5	NE	
8211/3: Tubular adenocarcinoma	997 (0.6)	81 (0.3)	0.42	0.34 to 0.54	55 (0.3)	0.45	0.34 to 0.59	88 (0.3)	0.55	0.44 to 0.69	<=5	NE	
8480/3: Mucinous adenocarcinoma	2911 (1.8)	554 (2.1)	1.23	1.12 to 1.35	503 (2.4)	1.5	1.36 to 1.66	471 (1.7)	1.17	1.06 to 1.29	15 (1.1)	0.69	0.43 to 1.13
8523/3: Infiltrating duct mixed with other types of carcinoma	5265 (3.2)	903 (3.4)	1	0.93 to 1.08	709 (3.4)	1.05	0.97 to 1.14	922 (3.4)	1.1	1.02 to 1.18	31 (2.2)	0.7	0.5 to 0.99
8507/3: Ductal carcinoma, micropapillary	607 (0.4)	122 (0.5)	1.26	1.03 to 1.54	101 (0.5)	1.17	0.95 to 1.45	118 (0.4)	1.02	0.83 to 1.26	<=5	NE	

©2020 Kong X et al. *JAMA Network Open*

Other	5314 (3.3)	1273 (4.7)	1.38	1.29 to 1.47	669 (3.2)	1.05	0.96 to 1.14	973 (3.5)	1.2	1.12 to 1.29	48 (3.4)	1.05	0.79 to 1.4

Abbreviations: NHW, Non-Hispanic White.

*Adjusted for age, SEER registry region, and year of diagnosis. Case-case OR = $(\%_{subtype}/\%_{Infiltrating\ Duct\ Carcinoma})_{subpop}/(\%_{subtype}/\%_{Infiltrating\ Duct\ Carcinoma})_{NHW}$.

©2020 Kong X et al. *JAMA Network Open*

eTable 4. Number and Percentage of Patients with Each T Stage of Breast Cancer by Race/Ethnic Group and Case-Case ORs for Each T Stage Compared With T1

Tumor Size (T Stage)	Non-Hispanic White No. (%)	Black			Asian/Pacific Islander			Hispanic White			American Indian/Alaskan Native		
		No. (%)	OR*	95% CI	No. (%)	OR*	95% CI	No. (%)	OR*	95% CI	No. (%)	OR*	95% CI
T1	99334 (61.2)	13099 (48.7)	Ref.	-	11673 (55.4)	Ref.	-	14166 (51.7)	Ref.	-	782 (55.7)	Ref.	-
T2	47397 (29.2)	9655 (35.9)	1.51	1.47 to 1.56	7273 (34.5)	1.2	1.16 to 1.24	9850 (36)	1.31	1.27 to 1.35	457 (32.6)	1.16	1.03 to 1.3
T3	9466 (5.8)	2368 (8.8)	1.84	1.75 to 1.94	1321 (6.3)	0.99	0.93 to 1.05	2156 (7.9)	1.29	1.23 to 1.36	98 (7)	1.15	0.93 to 1.42
T4	5993 (3.7)	1763 (6.6)	2.18	2.06 to 2.31	800 (3.8)	1.18	1.09 to 1.28	1212 (4.4)	1.45	1.35 to 1.55	66 (4.7)	1.5	1.16 to 1.94

Abbreviations: NHW, Non-Hispanic White.

*Adjusted for age, SEER registry region, and year of diagnosis. Case-case OR = $(\%_{subtype}/\%_{T1})_{subpop}/(\%_{subtype}/\%_{T1})_{NHW}$.

©2020 Kong X et al. *JAMA Network Open*

eTable 5. Number and Percentage of Patients with Each AJCC TNM Stage Group of Breast Cancer by Race/Ethnic Group and Case-Case ORs for Each Group Compared With Stage I

AJCC Stage Group (Stage)	Non-Hispanic White No. (%)	Black			Asian/Pacific Islander			Hispanic White			American Indian/Alaskan Native		
		No. (%)	OR*	95% CI	No. (%)	OR*	95% CI	No. (%)	OR*	95% CI	No. (%)	OR*	95% CI
Stage I	86220 (53.1)	10700 (39.7)	Ref.	-	10128 (48)	Ref.	-	11582 (42.2)	Ref.	-	648 (46.2)	Ref.	-
Stage II	52479 (32.3)	10339 (38.4)	1.54	1.5 to 1.59	7804 (37)	1.14	1.1 to 1.18	10656 (38.9)	1.33	1.3 to 1.37	506 (36.1)	1.2	1.06 to 1.35
Stage III	16911 (10.4)	4084 (15.2)	1.84	1.77 to 1.92	2369 (11.2)	1.06	1.01 to 1.11	3970 (14.5)	1.51	1.44 to 1.57	182 (13)	1.33	1.12 to 1.57
Stage IV	6241 (3.8)	1725 (6.4)	2.1	1.99 to 2.23	731 (3.5)	0.97	0.89 to 1.05	1119 (4.1)	1.27	1.18 to 1.36	65 (4.6)	1.39	1.07 to 1.8
UNK Stage	508 (0.3)	90 (0.3)	1.53	1.21 to 1.92	54 (0.3)	1.15	0.86 to 1.54	98 (0.4)	1.84	1.46 to 2.31	<=5	NE	

Abbreviations: NHW, Non-Hispanic White.

*Adjusted for age, SEER registry region, and year of diagnosis. Case-case OR = $(\%_{subtype}/\%_{Stage\ I})_{subpop}/(\%_{subtype}/\%_{Stage\ I})_{NHW}$.

©2020 Kong X et al. *JAMA Network Open*

eTable 6. Number and Percentage of Patients with Each Breast Cancer Tumor Site by Race/Ethnic Group and Case-Case ORs for Each Site Compared with Upper-Outer Quadrant of Breast

Tumor Site (PrimarySiteLabeled)	Non-Hispanic White No. (%)	Black			Asian/Pacific Islander			Hispanic White			American Indian/Alaskan Native		
		No. (%)	OR*	95% CI	No. (%)	OR*	95% CI	No. (%)	OR*	95% CI	No. (%)	OR*	95% CI
Upper-outer quadrant	57308 (35.3)	9337 (34.7)	Ref.	-	6789 (32.2)	Ref.	-	9248 (33.7)	Ref.	-	487 (34.7)	Ref.	-
Upper-inner quadrant	19965 (12.3)	3325 (12.3)	1.05	1 to 1.1	3041 (14.4)	1.26	1.21 to 1.33	3429 (12.5)	1.05	1.01 to 1.1	212 (15.1)	1.22	1.04 to 1.44
Lower-outer quadrant	12452 (7.7)	1953 (7.2)	0.98	0.93 to 1.03	1516 (7.2)	1	0.94 to 1.06	2006 (7.3)	0.97	0.92 to 1.02	97 (6.9)	0.89	0.71 to 1.11
Lower-inner quadrant	9093 (5.6)	1711 (6.4)	1.17	1.11 to 1.24	1140 (5.4)	1.12	1.05 to 1.2	1418 (5.2)	1.03	0.97 to 1.09	59 (4.2)	0.8	0.61 to 1.05
Central portion + Nipple	8159 (5)	1133 (4.2)	0.87	0.81 to 0.93	1196 (5.7)	1.42	1.33 to 1.52	1310 (4.8)	1.15	1.08 to 1.23	80 (5.7)	1.33	1.05 to 1.69
Overlapping lesion of breast	37215 (22.9)	5881 (21.8)	0.99	0.96 to 1.03	5104 (24.2)	1.15	1.11 to 1.2	6590 (24)	1.1	1.06 to 1.14	294 (21)	0.92	0.8 to 1.07
Axillary tail of breast	771 (0.5)	182 (0.7)	1.46	1.24 to 1.73	81 (0.4)	0.79	0.62 to 1	130 (0.5)	0.92	0.76 to 1.12	<=5	NE	

©2020 Kong X et al. *JAMA Network Open*

Breast, NOS	17396 (10.7)	3416 (12.7)	1.2	1.15 to 1.25	2219 (10.5)	1.04	0.99 to 1.1	3294 (12)	1.13	1.08 to 1.18	172 (12.3)	1.14	0.96 to 1.36

Abbreviations: NHW, Non-Hispanic White.

*Adjusted for age, SEER registry region, and year of diagnosis. Case-case OR = $(\%_{subtype}/\%_{Upper\text{-}outer\ quadrant})_{subpop}/(\%_{subtype}/\%_{Upper\text{-}outer\ quadrant})_{NHW}$.

©2020 Kong X et al. *JAMA Network Open*

eFigure 1. IRR of Breast Cancer Pathological Patterns by Race/Ethnicity Compared With NHWs

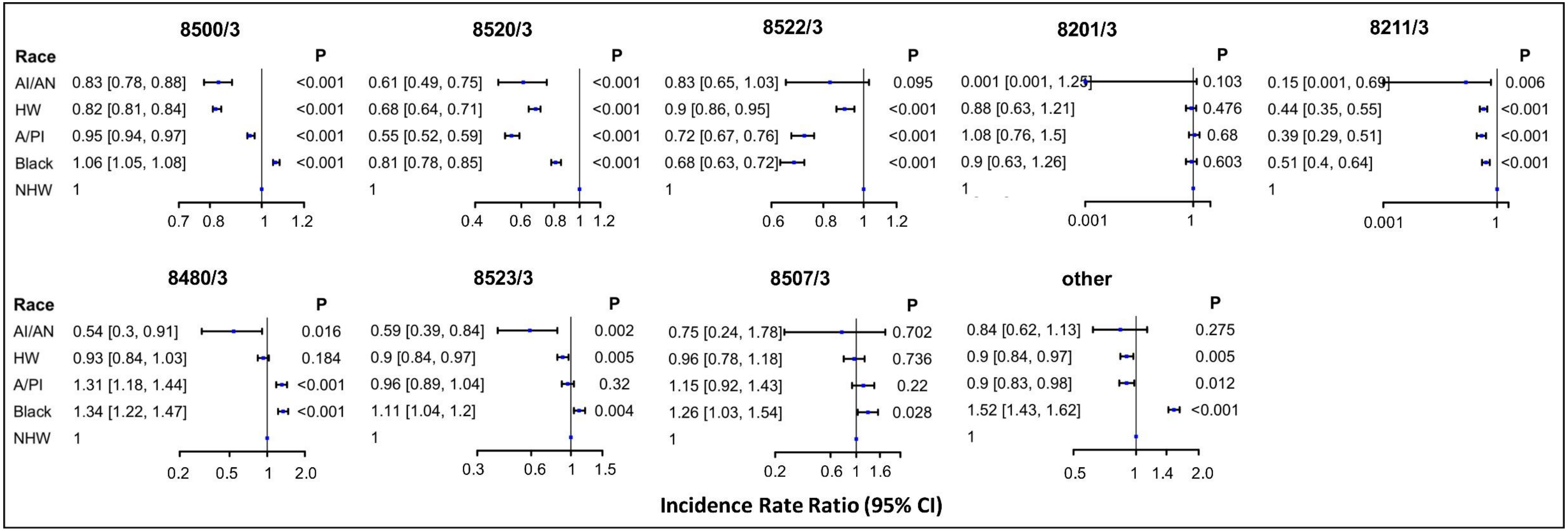

©2020 Kong X et al. *JAMA Network Open*

eFigure 2. IRR of Breast Cancer Tumor Locations by Race/Ethnicity Compared With NHWs

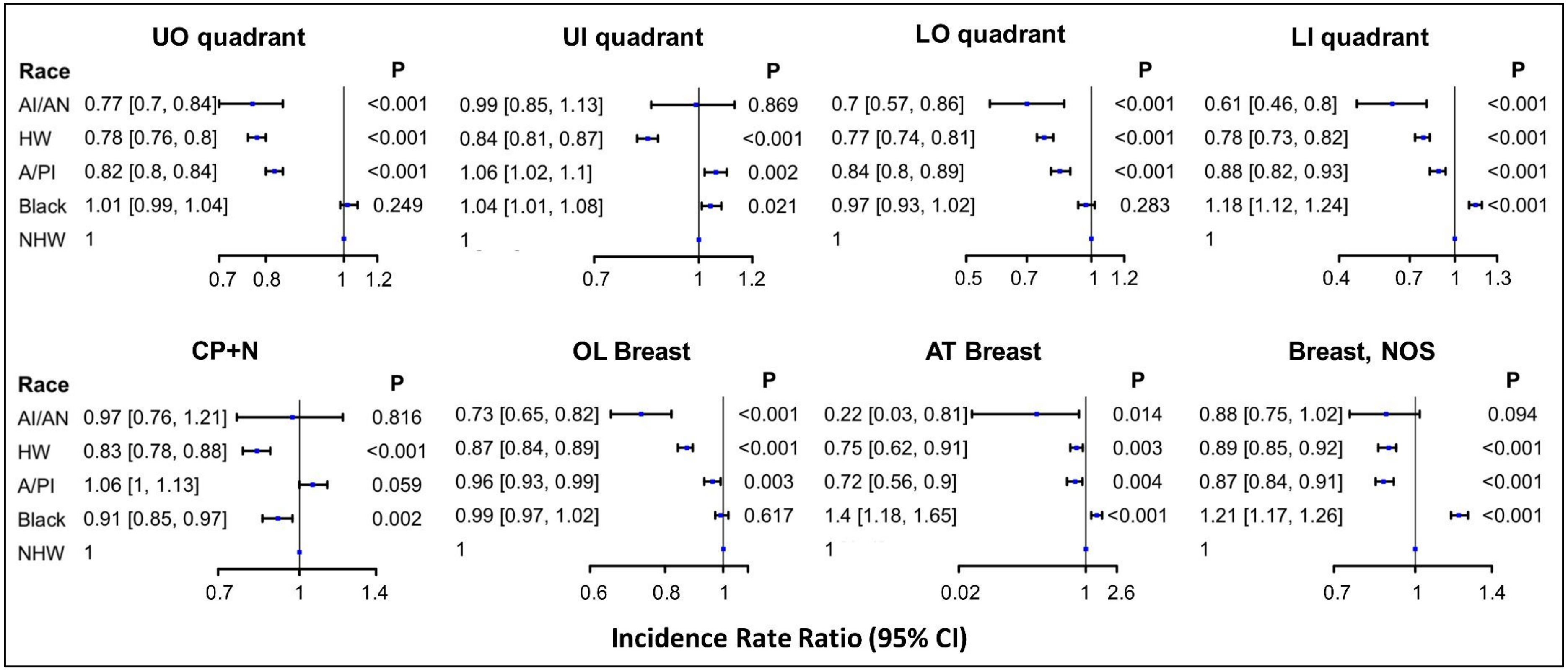

©2020 Kong X et al. *JAMA Network Open*
